# Africa vaccinating Africa: Pre- and post-COVID-19 perspectives, challenges, future prospects, and sustainability

**DOI:** 10.7189/jogh.13.03006

**Published:** 2023-01-27

**Authors:** Nicholas Aderinto, Elizabeth Oladipo, Oluwatimilehin Amao, Oyinkansola Omonigbehin

**Affiliations:** 1 LAUTECH Teaching Hospital, Ogbomoso, Oyo State, Nigeria; 2 Department of Medical Laboratory Science, Federal Neuropsychiatric Hospital, Lagos, Nigeria; 3 Department of Medical Laboratory Science, University of Abuja Teaching Hospital, Abuja, Nigeria

**Figure:**
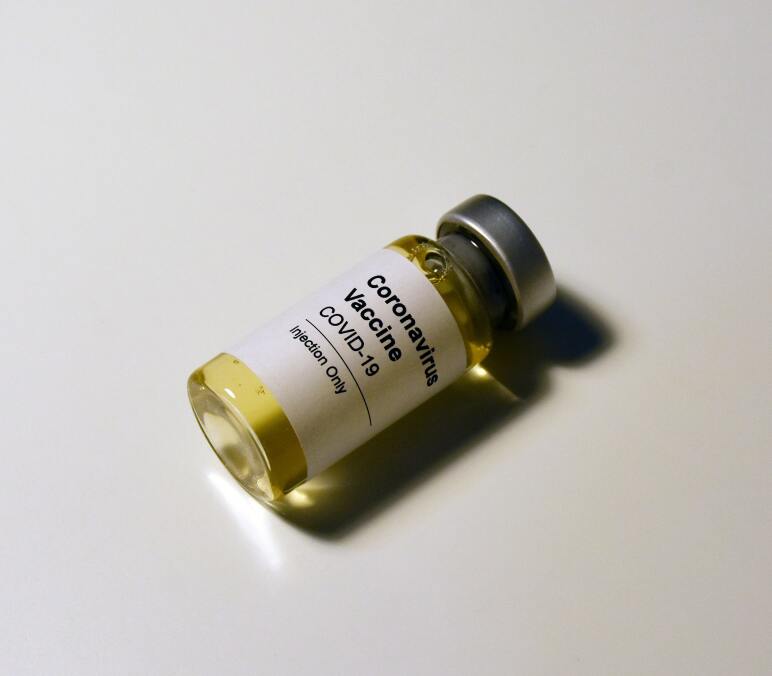
Photo: Rolling out hope: Africa’s vaccination efforts gather momentum in the fight against COVID-19. Source: created by Hakan Nural, free to use under Unsplash license (available at: https://unsplash.com/photos/Z2n-r7pg6kM).

The 2016 Global Burden of Disease Study [1] indicated that communicable diseases declined as a major cause of mortality over the previous ten years. A significant amount of this decline was due to decreases in major causes of worldwide mortality, such as HIV/AIDS, malaria, tuberculosis, and diarrheal illnesses. However, infectious diseases continue to be the main cause of death in parts of Africa and Asia, particularly in children under five years of age [2]. Sub-Saharan Africa and South Asia alone accounted for 44.4% and 24.8% of all child fatalities in 2016, respectively [3]. Additionally, diseases like malaria and invasive non-typhoidal salmonella (iNTS), also known as diseases of poverty, seem to be virtually wholly restricted to Africa [4].

Even though several African nations fell short of the Millennium Development Goals (MDGs), they made incredible progress, and the region's health outcomes significantly improved. The prevalence of HIV/AIDS, for instance, has been reported to have stabilized in several African nations [5,6]. Due to the significant potential for development, Africa also has the fastest economic growth globally [7]. Contrary to other emerging regions, local pharmaceutical production, notably for protein-based vaccines and biopharmaceuticals, is constrained, rising slowly and contributing to an expanding trade deficit and the poor availability of necessary medications [8]. Consequently, specific regional development organisations have started working together to expand the local production of pharmaceuticals in Africa as a part of their plans to boost the population's health and the continent's economy. For example, the East African Community (EAC) unveiled its Regional Pharmaceutical Plan of Action (2017-2027) [9]. The Southern African Development Community (SADC) amended its Pharmaceutical Business Plan [10]. This move is crucial because one of the challenges of local African vaccine production is the lack of competent national medicine regulatory authorities (NMRAs). South Africa, Kenya, Egypt and Morocco have comparable programs. These policies acknowledge, among other things, that domestic pharmaceutical production is a strategic requirement and a viable potential for growth because it can be less expensive than imports and would improve the trade balance [10]. For instance, Nigeria and Ethiopia could reduce their trade deficit by up to US$200 million annually if domestic pharmaceutical manufacturing increases by about two to three times [1]. The techniques cite cost-effective production as a successful tactic for enhancing poorer people's access to reasonably priced, high-quality medications.

Vaccination is one of the most significant medical innovations ever; it has drastically increased life expectancy, decreased mortality, and contributed to economic growth [2]. However, Africa is lagging in recognizing the potential for immunisation to lessen the disease burden. The Global Alliance for Vaccines and Immunization (GAVI) procures most vaccines in Africa [3], yet despite its efforts, it is unclear how to maintain such vaccine distribution and whether or not countries will be able to fund the introduction of new vaccines after leaving GAVI [4]. As African countries cannot be under GAVI forever, it is important to consider this future reality as efforts are made for local vaccine production. Even though economic cost-benefit analysis is just one of many factors which should be considered when determining health priorities [5], program cost will probably be the only deciding factor for the introduction of vaccines in most African nations [6]. Regarding vaccine procurement, cold chain capability, and programmatic logistics, a country's immunisation program will cost more as more vaccines are introduced there [7]. For instance, Ethiopia presently receives US$100 million from donors out of the US$150 million it spends annually on vaccine purchases [8]. After leaving, the Ethiopian government is anticipated to increase the budget allotted for acquiring vaccines to maintain its immunisation program [9]. Many nations, like India, Nigeria, and Thailand, have switched their attention from importing new vaccines with GAVI backing to domestic production options.

Manufacturers with headquarters in Egypt, Morocco, Senegal, South Africa, and Tunisia account for under 1% of the vaccinations in Africa [2]. On April 13, 2021, African leaders agreed on an ambitious plan to construct facilities and fund research and development to increase the proportion of vaccines manufactured in Africa from 1% in 2021 to 60% by 2040. Moreover, the African Development Bank has promised to finance at least two technology platforms worth US$400 million for vaccine production, and the Africa Center for Disease Control has plans to establish five new vaccine manufacturing facilities across the continent. These developments suggest that the continent is moving closer to developing its vaccine industry. The research and production of vaccines in Africa should be supported by technological innovation, such as whole-genome sequencing [1], to offer real-time knowledge on the biology and evolution of infectious organisms and lower production costs.

## VACCINE PRODUCTION IN THE PRE-COVID-19 ERA - WHAT WAS THE STATE BEFORE THE PANDEMIC?

Before COVID-19, there was widespread disrespect for vaccination throughout Africa. It was estimated that measles and smallpox claimed millions of lives. Millions of lives are saved annually through vaccination, considered one of the most cost-effective methods of avoiding disease and mortality. A remarkable example of coordinated international action in the fight against microbial invaders was the elimination of smallpox. The expansion of vaccination campaigns against measles and poliomyelitis, alongside their eradication from many areas, additionally prove that vaccination is one of the most effective public health measures. Until recently, just ten vaccine producers spread over five African nations – Egypt, Morocco, Senegal, South Africa, and Tunisia – could engage in local vaccine production. Due mainly to a deficiency of local scientific competence as well as flaws in the commodity supply chain, most of these countries have engaged in so-called fill-and-finish packaging and labeling with very little upstream development of antigen formulations [2,3]. The differences in COVID-19 vaccine coverage worldwide highlighted the inequities in global health care. Also, several low and middle-income (LMICs) African countries lack the knowledge, equipment, logistics, and monetary aid to mass-vaccinate their communities [4].

## POST-COVID-19 PERSPECTIVE - HOW HAS THE EMPHASIS ON VACCINE PRODUCTION IN AFRICA CHANGED?

The World Health Organization reported that Africa is falling behind other continents in the race to vaccinate its population following the discovery of the coronavirus disease and the introduction of vaccination.

The creation and distribution of COVID-19 vaccines provided some respite while also posing a new problem: unequal access to COVID-19 vaccines worldwide [5]. As of December 31, 2021, only 8% of Africans received all recommended vaccinations, compared to more than 70% of the population of affluent nations [6]. As a result of its dependence on outside producers due its inability to expand its capacity to produce sufficient doses of vaccines, Africa is disproportionately impacted by vaccine nationalism, stockpiling, and low economic status [7].

The COVID-19 vaccine has been produced in 413 million doses as of March 3, 2021 [8]. China leads the world in production with 141.6 million doses, followed by the United States with 103 million doses. India produced 42.4 million, compared to 70.5 million produced by Belgium, Germany, and other countries [8]. The pandemic has caused more damage, as seen by the rise in confirmed cases and fatalities, despite delayed spread to Africa [9].

COVID-19 vaccines were needed urgently, shortening the time needed for vaccine development and increasing the accessibility to potent technology. Advancements in immunotherapy and the new technologies fast-tracked for COVID-19 (RNA vaccines, viral vectors, and protein-based vaccines with potent adjuvants) may provide solutions for some of the contemporary society's most pressing problems, including emerging infections, AMR, chronic diseases, and cancer [10].

## CHALLENGES AND FUTURE PROSPECTS

Despite having the most significant rate of infectious illness mortality, Africa has been surprisingly incapable of providing the vaccinations necessary to lower mortality, increase life expectancy, and spur economic progress [1]. Expanding vaccine production capacity in Africa has been hampered by biological processes and their inherent inability to produce vaccination batches with uniform qualities and characteristics. Consequently, transferring technologies and production methods to African facilities is challenging. While India is an excellent example for private producers, Brazil and Cuba are fantastic learning examples of vaccine production setup by governmental institutions [3]. Through the United Nations Children's Fund (UNICEF) and the Pan American Health Organization (PAHO), these nations committed to developing or creating their biopharmaceutical manufacturing capability, initially focusing on domestic needs and then expanding to supply worldwide markets [4]. Despite the difficulties with technology transfer, this development became a significant source of income. The anticipated population rise and subsequent market expansion increase Africa's chances of success. Ethiopia, the Democratic Republic of the Congo, the United Republic of Tanzania, and Uganda will be among the twenty most populous nations in the world in 2100 [2].

Additionally, the East African region leads the continent's economy, which is expanding steadily at a rate of 5%-6% (as reported by the EAC) [3]. The development, maintenance, lead time, production facilities, equipment, life cycle management, and product portfolio management of vaccines are some unique problems that must be overcome [7]. To ensure a vaccine's long shelf life in the market, we stress the significance of a reliable and stable manufacturing process and continuous component supplies over many years. These industries should carefully consider funding the manufacturing of vaccines in Africa. Failure to manage these risks may result in pricey product recalls, market restrictions, and penalties if a business violates supply agreements. According to this theory, the choice of manufacturing technologies significantly influences the success of vaccine production, particularly given the environment now present in Africa. Regarding process stability and maintenance, life cycle, and lead time, the technologies for vaccine production, particularly the expression systems, are crucial to the cost of production. There are various expression platforms, each with different yield potential, and some require intricate development.

It is important to remember that the structure of vaccine markets in Africa is the main barrier. Without the dedication and backing to purchase vaccines made in Africa. Building a sustainable enterprise that can produce vaccines on a wide scale will always be a challenge. Vaccine production is complicated, with significant financial outlays and long-term planning. To focus on the main problem, such as creative financing, meeting current global policy standards, and enabling local and regional regulatory capacity to crucial technical elements like skill development, product development partnerships, good manufacturing practice design and establishment, there must be a collaboration with many partners. To sum up, the current COVID-19 pandemic has been eye-opening, offering an excellent opportunity to act on existing suggestions and discussions to facilitate increased vaccine production in Africa, immunisation against childhood diseases, and control of outbreaks of highly infectious diseases.

## References

[1] GBD 2016 Causes of Death Collaborators Global, regional, and national age-sex specific mortality for 264 causes of death, 1980–2016: a systematic analysis for the Global Burden of Disease Study 2016. Lancet. 2017;390:1151-210. 10.1016/S0140-6736(17)32152-9 28919116 PMC5605883

[2] Andre FE Booy R Bock H Clemens J Datta S John T Vaccination greatly reduces disease, disability, death and inequity worldwide. Bull World Health Organ. 2008;86:140-6. 10.2471/BLT.07.040089 18297169 PMC2647387

[3] Schütte C Chansa C Marinda E Guthrie TA Banda S Nombewu Z Cost analysis of routine immunisation in Zambia. Vaccine. 2015;33:A47-52. 10.1016/j.vaccine.2014.12.040 25919174

[4] Plotkin S Robinson JM Cunningham G Iqbal R Larsen S The complexity and cost of vaccine manufacturing – An overview. Vaccine. 2017;35:4064-71. 10.1016/j.vaccine.2017.06.003 28647170 PMC5518734

[5] Khan MI Ikram A Bin Hamza H Vaccine manufacturing capacity in low- and middle-income countries. Bull World Health Organ. 2021;99:479-9A. 10.2471/BLT.20.273375 34248217 PMC8243032

[6] Lawal L Aminu Bello M Murwira T Avoka C Yusuf Ma’aruf S Harrison Omonhinmin I Low coverage of COVID-19 vaccines in Africa: current evidence and the way forward. Hum Vaccin Immunother. 2022;18:2034457. 10.1080/21645515.2022.2034457 35240908 PMC9009957

[7] Rappuoli R Gregorio ED Giudice GD Phogat S Pecetta S Pizza M Vaccinology in the post−COVID-19 era. Proc Natl Acad Sci U S A. 2021;118:e2020368118. 10.1073/pnas.2020368118 33431690 PMC7826410

[8] Kaufmann JR Miller R Cheyne J Vaccine Supply Chains Need To Be Better Funded And Strengthened, Or Lives Will Be At Risk. Health Aff (Millwood). 2011;30:1113-21. 10.1377/hlthaff.2011.0368 21653965

[9] MacLennan CA Saul A Vaccines against poverty. Proc Natl Acad Sci U S A. 2014;111:12307-12. 10.1073/pnas.1400473111 25136089 PMC4151718

[10] Suárez-Álvarez A López-Menéndez AJ Is COVID-19 vaccine inequality undermining the recovery from the COVID-19 pandemic? J Glob Health. 2022;12:05020. 10.7189/jogh.12.05020 35604879 PMC9126303

